# Intergenerational associations in inhibitory control in *FMR1* families

**DOI:** 10.3389/fpsyt.2026.1820723

**Published:** 2026-04-28

**Authors:** Erin E. Hunt, Holley P. Arnold, Kaitlyn B. Cortez, Jane E. Roberts

**Affiliations:** 1 Department of Psychology, University of South Carolina, Columbia, SC, United States; 2 Carolina Autism and Neurodevelopment Research Center, Columbia, SC, United States

**Keywords:** executive functioning, *FMR1* conditions, genetic vulnerability, inhibitory control, intergenerational transmission

## Abstract

Inhibitory control (IC), the capacity to resist or suppress a dominant or automatic response, is a central component of self-regulation and executive functioning. Greater IC capacity in early development is also associated with a number of positive downstream outcomes. IC is highly heritable and susceptible to environmental influence in early development, highlighting a critical role of the family in transmitting and fostering this ability. Children with fragile X syndrome (FXS), an inherited neurodevelopmental disorder, and their mothers with the *FMR1* premutation (FXp) both evidence heightened vulnerability for IC deficits, warranting a need for a better understanding of familial IC associations in *FMR1* families. Informed by family systems and evocative gene by environmental interaction frameworks, the present cross-sectional, phenotypic study examined mother-child IC associations in early development in children with FXS and their FXp mothers in comparison to neurotypical (NT) mother-child dyads. We hypothesized that the association between maternal IC and child IC would be stronger for *FMR1* families given the compounding genetic and environmental vulnerability for lower IC demonstrated in extant research. Consistent with prior research, the present study documented IC deficits in children with FXS as well as nuanced processing differences FXp mothers compared to controls. Intergenerational IC associations were found for NT families. In contrast to our hypothesis, no associations were found between measures of maternal IC and child IC in *FMR1* families. Despite null findings for *FMR1* families, family-based interventions targeting IC deficits in these mothers and children could result in positive outcomes for the entire family system.

## Introduction

The study of self-regulation is vast, spanning multiple fields of research, including temperament, neuroscience, and neuropsychology literatures in recent decades. Across these fields and conceptualizations, various terms have been used to describe these distinct yet overlapping constructs (i.e., self-regulation, self-control, effortful control, executive functioning), with one central component consistently referred to as inhibitory control. Inhibitory control (IC) refers to the capacity to resist or suppress a dominant or automatic response (1), which is critical for learning and adaptive social-emotional and behavioral functioning (2). IC is also foundational to the development of broader self-regulatory functioning and executive functioning (3–5). Executive functions (EF) are higher-order cognitive skills supported by the prefrontal cortex and allowing for goal-directed behavior. Given the protracted development of the prefrontal cortex and its’ high sensitivity to environmental influence early in life, considerable individual differences in IC capacities emerge across infancy and early childhood. Impairments in IC are characteristic of several neurodevelopmental and genetic conditions, including *FMR1*-related conditions (i.e., fragile X syndrome, *FMR1* premutation). Despite extensive evidence for enhanced vulnerability for impaired IC across *FMR1*-related conditions (6–14), associations between mother and child IC have not been examined in this population. Informed by family systems and evocative gene x environmental interaction frameworks, the present study will examine phenotypic mother-child IC associations in children with fragile X syndrome (FXS) and their mothers with the *FMR1* premutation (FXp).

A family systems perspective holds that members of a family reciprocally influence one another’s behavior via both biological and social environmental mechanisms, and together, these interactions influence the broader family environment (15). This theoretical framework can also be extended to the study of IC development in early childhood (16, 17). The rapid maturation of the prefrontal cortex in the first few years of life supports the emergence and improvement in IC abilities (18), making early childhood a critical period for the development of IC. During this period, children and their developing brains are highly experience-dependent, rendering IC capacities highly susceptible to environmental influence. Further, the parent-child relationship serves as the primary social environment in which children develop these capacities (19, 20).

Social theories of EF development propose that processes such as IC are fostered by and influence interactions with a caregiver (20–22). Further, IC is intergenerationally transmitted via parenting behavior and interaction quality. For example, greater mother IC is associated with more sensitive parenting and interactions; high-quality interactions between mothers and children in early development bolsters child IC (23–27). Maternal EF has also been shown to influence growth rate in child EF via transactional associations with caregiving behaviors (26), with higher child EF eliciting higher quality parenting and higher quality parenting improving child EF (28). Evidence of associations between maternal and child IC being mediated by maternal emotional support (29) would further suggest that improving caregiver IC may have positive downstream effects on both interaction quality and child self-regulation outcomes.

Research has also consistently supported the high heritability of IC (30–32), indicating individual differences in IC are also strongly influenced by genes and gene-environment interactions (24, 88). A meta-analysis of self-control produced a heritability estimate of.60, which did not differ across gender or age (34). Given mothers and children share 50% of the same genes, it is likely that certain genes underly phenotypic characteristics in both the child and parent from whom the child inherited that gene (35). High concordance between maternal and child IC is evident as early as 24 months and stable across early childhood; these intergenerational associations have been demonstrated independent of IQ and controlling for verbal ability and maternal education in families of preschool-aged children using task performance measures of EF (31, 36). In contrast, evidence of an association between maternal EF and infant attention, but not infant EF, at 8 months is likely explained by a lack of valid measures and insufficient EF development at this young age (37).

Consistent with the transactional nature of parent-child interactions, Jaffee and Price (38) propose a concept of evocative genotype-environment interactions that references the “association between an individual’s genetically influenced behavior and others’ reactions to that behavior.” While evocative genotype-environment interactions may suggest compounding risk, it is also possible that children with genetic risk for IC deficits are more susceptible to the positive effects of environmental self-regulation supports, in accordance with a differential susceptibility approach (39). Consequently, a better understanding of parent-child IC associations in families at risk for IC impairment could inform the development of targeted interventions that benefit the entire family system.

FXS is an inherited neurodevelopmental disorder identified by an expansion of the CGG repeat sequence (i.e., >200 CGG repeats) on the *FMR1* gene on the long arm of the X chromosome. It affects approximately 1 in 4,000-5,000 males and 1 in 2,500-10,000 females (40, 41), with greater severity of problems seen in males with the condition. FXS is characterized by intellectual disability, and elevated rates of internalizing problems, externalizing problems, autism, ADHD, and anxiety (42–44). Given the location on the X chromosome, FXS is typically inherited from mothers with FXp (i.e., 55–200 CGG repeats). The prevalence of FXp in women in the general population has been estimated at roughly 1 in 151 women (45). Research has documented elevated rates of anxiety and depression in women with FXp relative to the general population (46, 47). Additionally, FXp mothers of children with FXS are at increased risk for negative health outcomes which may be attributed to both genetic risk and the stress of parenting a child with behavioral challenges (48, 49).

IC deficits are central to the FXS phenotype (6–9). Children with FXS exhibit lower parent-reported effortful control (EC), the broader temperament construct subsuming IC and attentional control, compared to neurotypical (NT) boys as well as a lack of improvement in EC across early childhood (50). These findings have been extended to IC, with lower IC evident in FXS compared to NT males by 24-months of age; lower levels of IC at 24 months-of-age and less improvement in IC with age also predicted greater expression of ADHD symptoms and ASD symptoms, respectively, at age 5 years (10). Lower IC in FXS males relative to children with other genetic syndromes and mental age-matched NT peers suggests IC deficits in FXS are not solely attributable to intellectual impairment (7, 51, 52). Atypical activation patterns in striatal and prefrontal brain regions underlying IC performance have also been documented in females with FXS, despite no differences in IC performance across FXS vs. NT groups (53). These findings suggest that individuals with FXS may engage alternative brain networks during IC. Other studies have indicated lower EF in females with FXS, controlling for IQ, and positive associations between X activation ratio (i.e., the proportion of cells with an unaffected *FMR1* gene on the active X chromosome) and EF, which highlights the centrality of EF deficits to the neurocognitive phenotype of females with FXS (54).

IC deficits also appear central to the neuropsychological phenotype of FXp women (11–13). However, some studies have failed to find IC differences (e.g., 33), possibly due to measurement or variable selection. For example, a previous study found that response latency, rather than errors, was a more sensitive index of IC deficits in FXp women (12); these authors postulated that response latency better reflects the efficiency of this cognitive process and the effort dedicated to achieving successful IC. Other studies determined that female premutation carriers performed worse on performance-based tests of IC compared to age- and IQ-matched controls (14). A higher proportion of errors on an ocular motor anti-saccade test of IC has also been associated with greater generalized behavioral dysregulation in this group (13). In terms of factors contributing to IC risk, premutation carriers with midrange CGG repeat lengths (i.e., 80–100 repeats), lower X activation ratio, and older age also demonstrate increased vulnerability for dysregulated IC according to both self-report and response latency on behavioral assessment measures (12, 55, 56).

FXp mothers of children with FXS also exhibit IC deficits, suggesting a familial component to this shared impairment. Parenting status may also influence the expression of IC deficits in these women. A study by Kraan et al. (56) determined that, after excluding mothers of children with FXS from the sample of FXp women, the IC performance of premutation carrier women no longer significantly differed from controls. These findings support an evocative gene x environment interaction model, with the heightened stress of parenting a child with disabilities and likely IC deficits, perturbing the genetic predisposition for impaired IC in premutation carrier mothers (57). More broadly, impaired IC has also been proposed as a neuropsychological marker of “probably caseness for mental disorder (i.e., ADHD-predominantly inattentive, social anxiety, depression)” in female premutation carriers (56), highlighting the potential for IC as an early risk marker and target for intervention.

### Current study

Genes that confer risk for specific impairments are often passed down from parent to child, making these family systems susceptible to vulnerabilities at multiple interacting levels. Despite extensive evidence for enhanced vulnerability for impaired IC across *FMR1*-related conditions, associations between mother and child IC have not been examined in this population. Preliminary work indicates that *FMR1* families with low child IC and high maternal depressive symptoms may be particularly vulnerable to dysfunctional parent-child interactions (58). Given naturalistic, parent-based approaches to intervention are becoming increasingly common for children with neurodevelopmental disorders, a better understanding of how IC operates within the parent-child dyad in *FMR1* families may elucidate multilevel behavioral targets for prevention, intervention, and treatment monitoring, which could result in positive outcomes for the entire family system. Further, interventions targeting IC could leverage the malleability and susceptibility of frontal lobe-supported processes to environmental input in early development and have an optimizing impact on subsequent neurodevelopmental trajectories. Thus, this study seeks to understand the relationship between maternal IC and child IC in *FMR1* and NT families and employs group-level phenotypic comparisons. Because of the compounding genetic and environmental vulnerability for lower IC, we expect the association between maternal IC and child IC will differ as a function of group, and therefore, anticipate the relationship between maternal IC and child IC to be stronger for *FMR1* families relative to NT families. Of note, examination of genotype-phenotype associations, though an important area of future research, is not within the scope of the present study. The present study does not leverage biological or genetic risk markers (e.g., CGG repeat length, X activation ratio, FMRP levels), but instead leverages genetic status (i.e., presence or absence of *FMR1* mutation) to differentiate groups. Further, the cross-sectional nature of the current study prevents claims or inferences regarding causality of behaviors within dyads.

## Method

### Participants

The present sample included 23 mother-child dyads with *FMR1*-related conditions, and 24 NT mother-child dyads. Of the FXS children with available data (*n* = 16), 68.8% evidenced mosaicism. This study leveraged extant data from longitudinal studies examining the early development of autism and anxiety in children with FXS (R01MH090194; R01MH107573; PI: Roberts) and supplemented with maternal IC data from mothers who responded to a follow-up email. Given preschool is a critical period for IC development, a cross-sectional sample was drawn from extant child data at approximately age 4 years matching groups on chronological age (*t* (45) = -0.36, *p* = .722).

Primary recruitment strategies for participation in the longitudinal studies included social media, community postings, and collaborations with other research groups throughout the country. Families of NT and FXS children were recruited via similar means, though recruitment efforts were more expansive for *FMR1* families given the rare incidence of these conditions. *FMR1* families were recruited from across the country, whereas participating NT families were recruited from the local area surrounding the university. Sample inclusion for this study stipulated a) full-term pregnancy (i.e., ≥37 weeks gestation), b) absence of medical conditions, c) English as primary spoken language, d) absence of personal or family history of ASD, ADHD, or intellectual disability, for NT participants, e) availability of child IC and developmental ability data, and f) only one parent-child dyad included per family. For multi-incidence families, children were selected to balance sex across groups, starting with FXS as the target group and matching the NT group accordingly. Genetic status (i.e., CGG repeat number) was confirmed via genetic analysis or medical records for children with FXS and their mothers. Given differences in genetic analysis methods used across medical institutions and differences in data availability across participants, integration or comparison of genetic data was not within the purview of the present phenotypic study.

### Measures

#### Child IC

The Inhibitory Control subscale of the Children’s Behavior Questionnaire (CBQ; 59) was used as a measure of parent-rated child IC. Parents rate 13 items, such as “Has difficulty waiting in line for something” and “Can easily stop an activity when s/he is told ‘no,’” on a 7-point Likert scale (1=extremely untrue, 7=extremely true), according to how well the item describes their child. Item scores are averaged to produce a total score ranging from 1-7, with higher scores indicating greater IC abilities. The CBQ has been validated for use with children with FXS (60).

#### Child developmental ability

The Mullen Scales of Early Learning, (MSEL ELC; 61) Early Learning Composite (*n* = 41) or Differential Ability Scales, 2^nd^ Edition (DAS-II; 62) General Conceptual Ability (*n* = 6) standard scores were included as estimates of developmental ability and covaried where indicated. Scores were drawn from either the concurrent (*n* = 45) or most recent (*n* = 2) assessment completed.

#### Internalizing and eternalizing symptom severity

Internalizing and externalizing symptom severity was assessed during their most recent assessment via the Child Behavior Checklist for Ages 1½-5 (CBCL (63)), a parent rating scale of emotional and behavioral functioning, which has been used previously with children with FXS (42, 64, 65). Internalizing and externalizing raw scores were included to describe the behavioral characteristics of this sample, with higher scores indicating more severe internalizing or externalizing symptoms.

#### ASD symptom severity

ASD symptom severity was assessed via the Autism Diagnostic Observation Schedule, Second Edition (ADOS-2 (66)), a play-based, semi-structured socio-behavioral assessment, and gold-standard tool for the diagnosis of ASD that has been extensively used to measure ASD symptoms in individuals with FXS (67–70). Calibrated severity scores (CSS) were included to describe the behavioral characteristics of this sample. CSS scores range from 1 to 10, with higher scores indicating more severe ASD symptoms.

#### Maternal IC

The Hayling Sentence Completion Task (71) was administered as a direct assessment measure of maternal IC. In Section 1 (S1) of the task, the examiner reads 15 sentences with a missing final word, and participants are asked to, as quickly as possible, provide a word which completes the sentence (sensible completion). Response latency was recorded for each sentence and summed for each section. The S1 latency score has been used in prior research as a measure of *response initiation* (72–74), with higher S1 latency scores reflecting poorer response initiation. In Section 2 (S2), the examiner reads a new set of 15 sentences with the final word missing and participants are asked to, as quickly as possible, provide a word which *does not* fit at the end of the sentence and is completely unconnected to the sentence (unconnected completion). In S2, responses for each sentence were assigned an error score based on the level of connectedness to the sentence and scores were summed. Higher S2 latency and S2 error scores represent poorer IC. Lastly, S2 - S1 latency scores were calculated and included in *post-hoc* analyzes as a purer measure of IC removing the confound of response initiation (73, 75). The Hayling task has been previously used with FXp adult females (12, 14, 56) and was chosen given flexibility of administration to reduce participation burden. Though temporal effects for maternal IC were not expected, given its stability in early to middle adulthood (76–78), correlations between maternal IC and age were examined in the present study.

#### Maternal intellectual quotient

The Wechsler Abbreviated Intelligence Scale (WASI; 79) is a standardized and widely used IQ measure. The FSIQ-2 score was incorporated as an index of maternal intellectual ability and included as a covariate where indicated.

### Procedures

Procedures were approved by the University of South Carolina Institutional Review Board and informed consent was obtained from all participating families. Assessments occurred in the child’s home or in a research lab setting. Mothers completed questionnaires, and direct assessments were administered by trained research staff according to standardized procedures. All mothers of NT and FXS children who participated in the longitudinal studies and met inclusion criteria were invited to participate in a follow-up assessment of their IC. Completion rate was 68%, with 47 of the total 69 mothers contacted agreeing to complete the follow-up maternal IC measure. Of the 22 families (12 *FMR1*, 10 NT) who did not respond to follow-up, 3 *FMR1* families had multiple children with FXS enrolled in the initial study which may be associated with greater stress burden and therefore reduced capacity for participation; however, 4 *FMR1* families who responded to follow-up also had multiple children with FXS. Comparison of extant maternal data does not suggest differences in maternal age for participating versus non-participating families, but does indicate differences in depressive symptoms, such that the mothers from the non-participating families reported more depressive symptoms during the initial study.

### Statistical analysis plan

SPSS statistical software was used for all statistical analyzes. Descriptive statistics were calculated for both groups (i.e., FXS, NT) of dyads. Groups were compared descriptively using Cohen’s *d*, with *d* ≥ 0.20, *d* ≥ 0.50, and *d* ≥ 0.80 interpreted as modest, moderate, and strong effect sizes, respectively (80). Data were examined for normality using data visualization and boxplots, and outliers were removed (e.g., >2.5 SD above or below the mean).

To examine the association between maternal IC and child IC, semi-partial correlations were calculated for each group, with maternal IC as the independent variable and child IC as the dependent variable. Child age, child developmental ability, child sex, maternal IQ, and maternal age were considered as covariates pending the strength of associations with primary variables (i.e., maternal IC, child IC) assessed via bivariate correlations; variables that were highly correlated with either primary variable were included as covariates. Given the potential inclusion of covariates, a semi-partial correlation approach was used to determine the *unique* variance in child IC explained by maternal IC. Semi-partial correlation values were compared across FXS versus NT groups; comparison across groups was descriptive in nature, given small sample sizes are associated with reduced power to detect significant effects and increase the likelihood of type II error. Significant semi-partial correlation values were squared to obtain effect size estimates (i.e., *R^2^*) representing unique variance explained in child IC by each maternal IC measure.

## Results

### Descriptive statistics

Descriptive statistics for both FXS and NT dyads are included in **Table 1**. There were strong group differences in child developmental ability (*d* = 3.42) and child IC (*d* = 1.11), with children with FXS demonstrating lower developmental ability and IC compared to the NT group. Additionally, children with FXS demonstrated higher ADOS severity scores (*d* = 1.29), greater CBCL internalizing problems (*d* = 1.31), and greater CBCL externalizing problems (*d* = 0.94) compared to NT children. In terms of maternal IC, FXp mothers demonstrated moderately longer S1 latency (*d* = -0.58) and no other differences were noted (*d* < 0.50). There were no other group differences in maternal descriptive variables (age and IQ *d* < 0.50).

**Table 1:** Demographic and descriptive characteristics.

Measures	FMR1 *M (SD)*	NT *M (SD)*	Cohen’s *d*
Child Variables			
*n*	23 (6 females)	24 (6 females)	
CA	4.52 (1.04)	4.42 (0.97)	-0.10
Developmental Ability	59.87 (13.35)	103.08 (11.93)	3.42
CBQ IC	3.52 (1.05)	4.64 (0.99)	1.11
CBCL-Internalizing Problems	11.86 (7.24)	4.26 (3.93)	1.29
CBCL-Externalizing Problems	16.76 (8.77)	9.04 (7.72)	1.31
ADOS-2 CSS	6.48 (2.75)	3.17 (2.37)	0.94
Mother Variables			
CA	41.35 (5.55)	40.42 (4.83)	-0.18
IQ	107.16 (14.37)	112.10 (7.57)	0.43
Hayling			
S1 Latency	21.38 (8.35)	17.12 (6.32)	-0.58
S2 Latency	33.71 (17.91)	36.14 (21.11)	0.12
S2 Errors	3.50 (3.35)	2.21 (2.73)	-0.42
S2 - S1 Latency	12.87 (16.66)	19.02 (20.53)	0.33

Child developmental ability and Mother IQ are standardized scores based on the normative sample of those measures (i.e., MSEL ELC; WASI-2 FSIQ2). Raw scores were used for Hayling S1 Latency, S2 Latency, S2 Errors, and S2 - S1 Latency.

*S1*, Section 1.

*S2*, Section 2.

*CA*, Chronological age in years.

*CBQ IC*, Children’s Behavior Questionnaire Inhibitory Control subscale, raw score.

*CBCL-Internalizing Problems*, Child Behavior Checklist Internalizing Problems subscale, raw score.

*CBCL-Externalizing Problems*, Child Behavior Checklist Externalizing Problems subscale, raw score.

*ADOS-2 CSS*, Autism Diagnostic Observation Schedule, Second Edition Calibrated Severity Score.

### Bivariate correlations

Child IC was significantly positively associated with child age (*r* (23) *= .*44, *p* = .037) and developmental ability (*r* (23) *= .*62, *p* = .001) for the FXS group only. Therefore, child age and developmental ability were included as covariates in semi-partial correlation analyzes. No other associations were found between child developmental ability, child age, maternal IQ, or maternal age with child IC or maternal IC.

### Semi-partial correlations

Semi-partial correlations were calculated for each group separately controlling for child age and developmental ability (**Table 2**). For the FXS group, there were no significant associations between any of the maternal IC measures (Hayling S1 latency, S2 latency, S2 errors, or S2 - S1 latency) with child IC. Maternal IC as measured by Hayling S2 latency (*sr* (24) = -.51, *p* = .015), and S2 - S1 latency (*sr* (24) = -.50, *p* = .017) was significantly negatively associated with child IC for the NT group only with longer response latency for S2 and greater latency difference between S1 and S2 associated with lower child IC. Hayling S2 latency and S2-S1 latency accounted for 26% and 25% of unique variance in child IC, respectively. There was also a trend towards greater S2 errors being associated with poorer child IC for the NT group (*sr* (24) = -.41, *p* = .058), with S2 errors accounting for approximately 17% of unique variance in child IC.

**Table 2:** Semi-partial correlations controlling for child age and developmental ability.

Measures	Child CBQ IC	
	FMR1	NT
Mother Hayling		
S1 Latency	.27	-.11
S2 Latency	.17	-.51*
S2 Errors	.12	-.41
S2 - S1 Latency	.03	-.50*

*p<.05. **p<.01.

Raw scores were used for Hayling S1 Latency, S2 Latency, S2 Errors, and S2 - S1 Latency.

S1, Section 1.

S2, Section 2.

*CBQ IC*, Children’s Behavior Questionnaire Inhibitory Control subscale, raw score.

## Discussion

The literature shows that IC is highly heritable and transmitted via social learning; however, no study to date has examined the association between maternal IC and child IC in *FMR1* families, for whom IC deficits are central to the behavioral phenotypes. A family systems approach to understanding intergenerational transmission of IC is beneficial to considering multilevel targets for intervention which may have cascading benefit for the entire family system. There is value in comparing intergenerational IC associations across groups with and without vulnerability for IC deficits as group differences may suggest multiplicative effects of environmental and genetic vulnerability, thereby flagging family systems who may benefit most from interventions targeting IC deficits. Although the present study did not directly assess genetic vulnerability at the molecular level, demonstration of family-level phenotypic differences in *genetically-defined groups* at heightened risk for IC deficits may have clinical implications that extend to other disorders characterized by elevated familial risk for executive dysfunction (e.g., ADHD, autism).

Consistent with extant literature documenting IC deficits in children with FXS, descriptive data indicate strong group differences in IC, with considerably lower IC observed in the children with FXS compared to NT children (7, 10, 51). IC deficits are a specific phenotypic characteristic seen in children with FXS and do not simply reflect intellectual disability. Examination of associations between child IC and developmental ability in the present study suggests these are related but distinct constructs and support that IC deficits explain group differences beyond developmental ability. FXp mothers demonstrated moderately slower response initiation (S1 latency) than mothers of NT children, whereas differences on inhibitory control measures were inconsistent and ambiguous. Evidence of slower response speed in FXp adult females relative to controls is variable, with some studies reporting slower (14, 81) and more variable (82) response speed in FXp females, and others reporting no group differences (83). Overall, findings are consistent with pronounced IC deficits in children with FXS and comparatively subtler IC differences in FXp mothers relative to NT dyads.

Unlike other studies documenting associations between lower IC and increasing age in FXp adult women (12, 84), the current study found no association between IC and age. This is likely due to this sample’s truncated age range in early to middle adulthood, whereas other study samples extend to older adulthood, a period in which decline in cognitive functions including IC is more likely to occur. Additionally, the current study did not examine the association between maternal IC and genetic indices (e.g., CGG repeat length, X activation ratio), such that it is possible that the current sample of FXp mother may not demonstrate the full range of genetic risk for IC impairment. Further, cursory comparison of participating versus non-participating FXp mothers indicated possible differences in depressive symptoms, therefore it is possible the current sample does not reflect the true psychosocial or behavioral variability seen in this condition.

The directionality of associations between maternal and child IC was in the anticipated direction for the NT group, with greater maternal IC (i.e., faster S2 and S2 - S1 latency, fewer S2 errors) associated with greater child IC. The magnitude of mother-child IC associations was also similar to other studies of familial EF (26, 31, 36). Contrary to our hypotheses, mother-child IC associations were not stronger for *FMR1* families, and we failed to find associations between any measure of maternal IC with child IC. Lack of associations between response latency and child IC for the FXS group is unsurprising, given a faster latency score for this group may be misleadingly interpreted as better IC if also associated with greater errors. Though true lack of concordance between maternal and child IC may underly null findings of intergenerational IC associations in the *FMR1* families, findings may also be explained by measurement-related factors which may differentially impact groups (e.g., measurement invariance, informant bias). Most notably, the IC measures used across generations differ in scope (i.e., parent-report versus performance-based task) and therefore represent related but not identical constructs. Though many available measures of executive functioning are not intended or appropriate for use across the lifespan, especially for use with young children (i.e., < age 5 years) with developmental and intellectual disabilities, future study involving homologous measures is necessary to further clarify the intergenerational IC associations.

Interestingly, the strongest association for the FXS group, though not statistically significant, was between S1 latency and child IC, with slower response initiation associated with better child IC. This may suggest that slower response initiation is actually advantageous in curtailing poor IC in FXp mothers. For example, during interactions with their children, slower response initiation may prevent reactive responding to child behaviors, thereby modeling appropriate self-regulation. Further, mitigated reactive responding may also allow mothers to respond more sensitively to child cues, which is known to be associated with better child EF (85, 86). Additionally, if FXp mothers are taking longer to respond to child cues, the child is afforded practice waiting for a response which may improve their IC over time. Consistent with the reciprocal nature of individual’s behaviors within a family system, as children develop greater IC, they also provide opportunities for parents to reduce reactive responding. Given non-significant associations, the interpretation and hypotheses above based on these findings are speculative in nature and do not fully explain or support evidence of *different* intergenerational processes across groups. Further, parenting or dyadic behaviors were not measured in this study, though this would be an important area of future study. The cross-sectional design also limits our ability to ascertain directionality of IC associations or possible influence by other familial or biological factors.

Although the direct contributions of genes and environment on mother-child IC associations were beyond the scope of the present study, the evocative gene x environment interaction model (38) is still helpful to contextualize the broader study of intergenerational transmission of IC. The present study considered that IC deficits are part of the shared behavioral phenotype of *FMR1*-related conditions, which are inherited via the X chromosome. However, IC abilities are not localized to just the X chromosome and are therefore influenced by multiple genes which are differentially expressed across generations. We cannot neglect to also consider that many of these genes influencing IC may have been inherited from the father, reflecting “parent-of-origin effects” (87). These considerations likely interact and ultimately impact mother-child IC concordance. Further, independent of the relative genetic contributions of each parent on child IC, the child’s social environment is also composed of multiple other sources of modeled IC beyond the mother, namely the father, siblings, or other primary caregivers. This may explain why the strength of mother-child IC associations are constrained to small or moderate effect sizes, even for families with known IC vulnerabilities (87). Future work may compare intergenerational IC associations in *FMR1* families to other groups with high familial risk for executive dysfunction (i.e., attention-deficit/hyperactivity disorder, autism spectrum disorder), including associations with behavioral symptomology. Additionally, incorporation of measures of dyadic behaviors or parenting in future studies of intergenerational IC will further demonstrate *how* IC is transmitted and functions within family systems; this has important clinical implications for the development of family-based interventions targeting IC.

### Limitations and future directions

The current study was not without limitations. Given small sample sizes, analyzes were likely underpowered to detect significant effects or intergenerational associations. However, it is unlikely that small sample size explains the null findings for the FXS group based on the limited strength of associations and associations in the unanticipated direction for this group. It is also important to acknowledge that the current sample of FXp women includes only mothers of children with FXS, and therefore findings may not generalize to the broader population of FXp women. Further, as described above, lack of examination of specific genetic or molecular factors within the *FMR1* group limits clarity of the generalizability of the current sample which may not encompass the full spectrum of genetic risk highlighted in previous studies of IC in FXp women. This study also had several measurement limitations; as mentioned above, the S2 - S1 latency score was considered *post-hoc* as a purer measure of maternal IC controlling for response initiation. Indeed, many EF tasks require multiple abilities, making it difficult to isolate single EF measures. Future studies of IC in FXp women may consider controlling for response initiation or processing speed or identifying alternative measures that reduce the confounding effect. Another measurement limitation of the present study is that mothers and children completed different measures of IC. Whereas other studies of parent-child IC associations have included older children who are able to complete the same IC measures that are sensitive for adults, the current sample included preschool-aged children, many of whom have developmental delays and therefore would not be able to complete traditional EF measures. Consequently, this may have contributed to limited associations. Rather, the present study leveraged a more global parent-rating of child IC and a performance-based measure of verbal inhibition for mothers. Differences in the type and scope of these measures may have further reduced conceptual similarity and limit interpretation of null findings.

### Conclusions

The present study findings corroborate and extend the broader literature indicating vulnerability for IC deficits in children with FXS. FXp mothers demonstrated differences, albeit nuanced, in processes involving inhibition. Despite the apparent differences in these groups compared to controls, this study failed to demonstrate intergenerational associations in IC within *FMR1* families. Null associations in *FMR1* families are juxtaposed with clear evidence of mother-child IC associations in families of NT children, which may be related in part to measurement and design limitations of the present study. Consequently, future research involving larger samples, leveraging genetic data, and employing more directly comparable executive functioning measures will be necessary to further understand intergenerational IC vulnerability in *FMR1* families. It is plausible that additional intervening or confounding variables are necessary in elucidating the complexity of mother-child IC associations in *FMR1* families which warrants future study. Given evidence of significant benefits of family-based interventions, especially for families of children with neurodevelopmental disorders, implementation of such interventions targeting parent and child IC, parenting behavior, and parent-child interaction quality should be considered to ameliorate the consequences of IC deficits in *FMR1* family systems.

## Data Availability

The raw data supporting the conclusions of this article will be made available by the authors, without undue reservation.
